# Supplementary material for: Causal Relationship between Aging and Anorexia Nervosa: A White-Matter-Microstructure-Mediated Mendelian Randomization Analysis
Source: Biomedicines. 2024 Aug 16;12(8):1874. doi: 10.3390/biomedicines12081874 (PMC11351342; doi:10.3390/biomedicines12081874)
Supplement: Supplementary file 1 [file biomedicines-12-01874-s001.zip › Supplementary Figure/Figure S8/Leaveoneout plot of MR analysis for TL to ubm-b-1535.pdf]

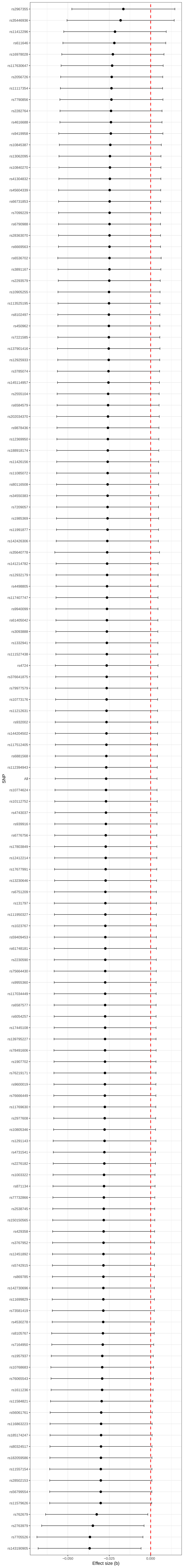
rs2967355
rs35446936
rs11412296
rs611646
rs16978028
rs117630647
rs2056726
rs11117354
rs7790856
rs2282764
rs4616688
rs9419958
rs10845387
rs13062095
rs10840270
rs41304832
rs45604339
rs66731853
rs7099229
rs6790988
rs28363070
rs6669563
rs6536702
rs3891167
rs2293579
rs10905255
rs113525195
rs8102497
rs450962
rs7221585
rs137901416
rs12925933
rs3785074
rs145114957
rs2555104
rs6584579
rs202034370
rs9878436
rs12369950
rs188918174
rs11426156
rs11085072
rs80116508
rs34550383
rs7209057
rs1985369
rs11991877
rs142426306
rs35640778
rs141214782
rs12932179
rs4498805
rs117407747
rs9940099
rs61405042
rs3093888
rs1332941
rs111527438
rs4724
rs376641875
rs79977579
rs10773176
rs11212631
rs932002
rs144204502
rs117512405
rs6881568
rs112394943
All
rs10774624
rs10112752
rs4743037
rs939916
rs6776756
rs17803849
rs12412214
rs17677991
rs13230646
rs6751209
rs131797
rs111950327
rs1023767
rs59409453
rs61748181
rs2230590
rs75664430
rs9955360
rs117034449
rs6587577
rs6054257
rs17445108
rs139795227
rs78491606
rs1907702
rs76219171
rs9600019
rs76666449
rs11769630
rs2977608
rs10805346
rs1291143
rs4731541
rs2276182
rs1003322
rs871134
rs77732866
rs2538745
rs150150565
rs429358
rs3767952
rs12451892
rs5742915
rs869785
rs142730696
rs11699829
rs73581419
rs4530278
rs8105767
rs7164950
rs1957937
rs10768683
rs76065543
rs1611236
rs11584821
rs56061761
rs116863223
rs185174247
rs80324517
rs182059586
rs11557154
rs28502153
rs56799554
rs11579626
rs762679
rs2763979
rs7705526
rs143190005
SNP
-0.050
-0.025
0.000
Effect size (b)
